# Advancing Social Impact in the Fight Against Antimicrobial Resistance: Lessons from the Infection Diagnosis Workshop

**DOI:** 10.3390/antibiotics15010064

**Published:** 2026-01-07

**Authors:** Thomas Mayers, C. Kiong Ho, Yuri Ushijima, Le Thuy Thi Nguyen, Le Quang Luan, Nguyen Van Thuan, Osamu Ohneda, Kazuya Morikawa

**Affiliations:** 1 Medical English Communications Center, Institute of Medicine, University of Tsukuba, Tsukuba 305-8575, Japan; mayers@md.tsukuba.ac.jp; 2 Department of Infection Biology, Institute of Medicine, University of Tsukuba, Tsukuba 305-8575, Japan; ushijima-yu@md.tsukuba.ac.jp; 3 Biotechnology Center of Ho Chi Minh City, Ho Chi Minh City, Vietnam; lenacns2005@gmail.com (L.T.T.N.); lequangluan@gmail.com (L.Q.L.); 4 School of Biotechnology, International University, Vietnam National University, Ho Chi Minh City, Vietnam; nvthuan@hcmiu.edu.vn; 5 Laboratory of Regenerative Medicine and Stem Cell Biology, Institute of Medicine, University of Tsukuba, Tsukuba 305-8575, Japan; oohneda@md.tsukuba.ac.jp

**Keywords:** antimicrobial resistance (AMR), One Health, educational program, medical science, social impact

## Abstract

Background/Objectives: Antimicrobial resistance (AMR) is a major global health threat that reduces antibiotic effectiveness and increases healthcare burdens. Countries in the Asia–Pacific region face a particularly high AMR burden, necessitating international collaboration, education, and practical training to combat this growing crisis. This study describes the design, implementation, and educational outcomes of the Infection Diagnosis Workshop, a short-term international program primarily targeting undergraduate medical sciences students that integrates AMR-focused hands-on clinical microbiology training and lectures, alongside cross-cultural collaboration and scientific English communication. Methods: The Infection Diagnosis Workshop was implemented as a four-day program combining lectures with hands-on laboratory activities. Training emphasizes the detection and analysis of antibiotic-resistant bacteria through environmental sampling, bacterial culturing, phenotypic and genotypic resistance detection, and species identification, core components that have remained consistent since the workshop’s establishment. Students also attended lectures on AMR science, global impact, and management strategies. Group discussions and collaborative tasks encouraged interdisciplinary learning. A thematic analysis of student feedback essays from previous workshop cohorts was conducted to identify key concepts, learning outcomes, and shared experiences. All participants provided informed consent for the use of their written feedback. Results: Thematic analysis revealed key learning outcomes categorized into three themes: (1) Knowledge, Awareness, and Technical Skills; (2) Cultural Understanding and Cross-Cultural Collaboration; and (3) English Language and Communication Skills. Students reported increased AMR knowledge, improved laboratory proficiency, enhanced cultural adaptability, and greater confidence in English communication. They also expressed a deeper appreciation for interdisciplinary and international approaches to AMR. Conclusions: The Infection Diagnosis Workshop effectively integrated practical laboratory training with international and cross-cultural engagement. The program strengthened student competencies and contributed to building global partnerships essential for combating AMR.

## 1. Introduction

Antimicrobial resistance (AMR) poses a significant threat to global public health, as it diminishes the effectiveness of antibiotic treatments for common infections, leading to prolonged illness, increased healthcare costs, and higher mortality rates [1]. Resistant bacteria can spread quickly, making even routine medical procedures, such as surgeries or chemotherapy, much riskier. In 2019, AMR was responsible for an estimated 1.27 million deaths globally, with 4.95 million deaths associated with drug-resistant infections [1,2]. According to data from the Global Antimicrobial Resistance and Use Surveillance System (GLASS), resistance to critical antibiotics such as third-generation cephalosporins for *Escherichia coli* is around 36% (interquartile range [IQR] 33.4–77.8), while methicillin-resistant *Staphylococcus aureus* (MRSA) in bloodstream infections has a global median of about 12% (IQR: 6–26%) [3,4], findings that strongly demonstrate the need for international collaboration and stricter antibiotic usage policies.

The Asia Pacific region, which accounts for two-thirds of the global population, is regarded, according to Yam et al., as a critical “hotspot” for the rise and proliferation of AMR [5]. Within this region, Southeast Asian countries, which have a high incidence of infectious diseases, are of particular concern [5,6]. In Vietnam, the country of focus in the current paper, the burden of AMR is particularly severe, with 14,300 deaths directly attributable to AMR, making it a more significant cause of death than diseases like diabetes or respiratory infections [2,7]. Indeed, Vietnam has one of the highest antibiotic resistance rates in Asia, with studies showing that 80% of *Streptococcus pneumoniae* isolates in rural preschool children were multidrug-resistant [7]. In developed countries within Asia, such as Japan, antibiotic resistance rates are somewhat lower but still a significant issue. Japan has implemented various antimicrobial stewardship programs [8,9,10], but increased resistance rates for hospital-acquired infections like *Klebsiella pneumoniae* are a growing concern [11].

Infectious diseases and drug-resistant bacteria do not respect borders, making it crucial for countries to work together to share knowledge, data, and resources to combat AMR [5]. Coordinated efforts can lead to the development of novel strategies for AMR prevention and treatment, and possibly innovation in the development of new antibiotics and alternative therapies. In this context, international exchange programs for healthcare and medical science students can play a vital role in this process by exposing them to diverse healthcare systems, practices, and challenges. In our experience of overseas exchange programs, we have found that these programs encourage the exchange of ideas, promote cultural understanding, and help students develop a global perspective on health issues [12,13,14]. By participating in such programs, students not only gain valuable technical skills but also become ambassadors of collaborative healthcare and gain a vision for contributing healthcare from a more international perspective.

Because education is a critical component of a sustainable approach to combating this crisis [15], faculty members of the Institute of Medicine, University of Tsukuba, initiated the Infection Diagnosis Workshop. The institute has a long history of promoting educational and research collaborations with Southeast Asian countries and has initiated multiple capacity-building educational programs designed to develop ties with partner universities and institutions, including courses in bioinformatics, gene editing courses, and molecular biology.

The Infection Diagnosis Workshop, which has been conducted since 2017 at partner institutes of the University of Tsukuba in Vietnam, brings together experts and participants to focus on this critical topic of AMR. The workshop curriculum focuses on the identification of drug-resistant bacteria and was primarily designed by the University of Tsukuba’s Bacteriology Laboratory, which has conducted extensive research in this field over many years. Furthermore, a graduate who completed her doctoral training in this laboratory went on to establish drug-resistant bacteria research capacity at the Biotechnology Centre of Ho Chi Minh City (HCMC), supporting the long-term sustainability of the workshop as a collaborative, research-based educational initiative in Vietnam.

The workshop provides both theoretical and hands-on training in detecting and analyzing antibiotic-resistant bacteria. Participants engage in a series of laboratory-based activities, including sample collection, culturing, phenotypic and genotypic detection of antibiotic resistance, and identifying bacterial species. The workshop aligns well with the One Health concept, which promotes the idea of the interconnectedness of human, animal, and environmental health [15,16,17,18]. By emphasizing AMR and incorporating activities like environmental sample collection and resistance pattern analysis, the workshop encourages a multidisciplinary perspective on AMR, emphasizing the need for collaborative, cross-sectoral solutions.

Unlike Japan, in Vietnam and neighboring countries, common antibiotics are effectively available over the counter without prescription and are often used casually [19,20]. Consequently, issues such as the transmission and proliferation of drug-resistant bacteria are more likely to occur and are more severe than in Japan [19,20]. These challenges are driven by various factors, including the use of antibiotics in livestock and nosocomial infections in urban hospitals [19,20]. In addition, the international spread of resistant bacteria and the resistance genes from Southeast Asia to other regions via livestock, meat products, and human movement represents a growing global concern [21]. The objective of the Infection Diagnosis Workshop, therefore, is to enable students to experience these realities firsthand and to provide experiences of working across borders and disciplines. This is achieved by engaging participants in addressing the global challenge of AMR through the application of insights from their respective specialist fields. This is essential, as the participants bring unique perspectives and serve as key stakeholders in the fight against AMR, enriching dialogue and driving innovative, context-sensitive solutions.

This alignment with the One Health concept emphasizes the importance of a holistic, multi-dimensional approach to AMR, which has implications for both human health and global ecosystems and has ramifications across healthcare, agriculture, and multiple sectors and industries [15,16,17,18,21]. In this context, attendees of the Infection Diagnosis Workshop not only gain technical expertise through lectures and collaborative exercises but also actively participate in discussions and presentations on the global challenge of managing AMR. By sharing their experiences and skills, they can offer potential solutions to this pressing issue. Since 2024, the Infection Diagnosis Workshop has been incorporated into the university’s Social Impact Project as part of the University of Tsukuba’s efforts to promote cross-cultural, experiential learning and global health education [22]. The Social Impact Project, launched by Japan’s Ministry of Education, aims to cultivate globally competent professionals by supporting international collaborative learning, student mobility, and the development of inclusive, multicultural educational environments [23]. The purpose of this report is to present a detailed explanation of the delivery of the workshop as it was held in Vietnam in 2024. This report will include an exploration of the outcomes and insights gained from the workshop through a thematic analysis of the feedback provided by participating students.

## 2. Materials and Methods

### 2.1. Description of the Course

#### 2.1.1. Organizers, Teaching Faculty, and Participants

The Infection Diagnosis Workshop is primarily organized and taught by faculty of the Department of Infection Biology, at the University of Tsukuba, with the assistance of faculty of partner universities in the host country, many of whom are alumni of the University of Tsukuba. Since the language of instruction of the workshop is English, a scientific English instructor from the University of Tsukuba provides teaching to support effective and enthusiastic communication and presentation.

Regarding student selection, initially, applicants were assessed by multiple faculty members based on written statements of motivation and academic goals, English language proficiency scores, and grade point average (GPA). In later iterations of the workshop, following the emergence of generative artificial intelligence (AI) tools, written statements were replaced with short video submissions (3–5 min) in which applicants described their motivation for participation and anticipated contributions to the course. Selection continued to be conducted by multiple faculty members, based on evaluation of the submitted materials and GPA. On average, the workshop can accommodate approximately 30 participants, who are divided into several groups. Each group comprises a combination of participants from Japan and the host country, along with one graduate student and/or teaching staff from the partner institute or university.

Workshop participants from Japan were undergraduates (in their early 20s) recruited from diverse backgrounds and disciplines across the University of Tsukuba, with some graduate students from the medical sciences serving as teaching assistants (TAs). In total, 86 University of Tsukuba students have participated in the workshop, 66 women and 20 men, including both Japanese (*n* = 72) and international students (*n* = 14). Participants are drawn primarily from medical sciences, with others joining from various other disciplines, including medicine, agrobiological sciences, engineering, international studies, and art.

Participants from the host country are recruited from an open call posted on our website, social media, and promoted through partner institutions in Southeast Asian countries. In total, 139 participants from the host country have joined this workshop. While most of these participants have been university students, a number of professionals including researchers, laboratory technicians, medical doctors, and pharmacists have joined the workshop.

#### 2.1.2. Pre-Workshop Assignment

Prior to the workshop, all participants were asked to review the laboratory manual and pre-learning materials to ensure a shared foundational understanding. Students from the University of Tsukuba were encouraged to research the biological and social background of AMR, including associated challenges, economic impacts, policy responses, emerging technologies, and treatment developments.

#### 2.1.3. Workshop Location, Facilities and Safety Protocol

The workshop must be held in laboratory facilities that strictly adhere to safety and technical standards for handling live and frequently pathogenic bacteria. The laboratories must be equipped with essential microbiological tools necessary for practical training in bacterial culturing. Key equipment includes biosafety cabinets, autoclaves for sterilization, specialized incubators for bacterial culture, PCR thermal cyclers, agarose gel electrophoresis setups, and microscopy stations for Gram staining and bacterial identification. Given the nature of the work with live pathogenic bacteria, strict safety protocols were observed throughout the course. In 2024, the workshop was held in the modern, well-equipped laboratories and teaching facilities at the Biotechnology Center of Ho Chi Minh City.

#### 2.1.4. Course Structure and Content

The workshop is held over a four-day period, with time allotted before the course for collection of bacterial samples. In the past, most of the samples have been isolates from a Vietnamese hospital; however, the inclusion of samples collected from the environment began in 2019, when samples were gathered from the Mekong Delta. In 2023, bacterial sampling was performed in Ho Chi Minh City and at a small farm in the rural outskirts of the city. In 2024, the students had the opportunity to gather samples in the historic city of Hoi An, a UNESCO World Heritage Site. Students were given swabs and asked to collect samples from the environment, especially from places that they suspected may be contaminated with *Staphylococci* and/or Gram-negative bacteria such as *E. coli*. In addition to the laboratory activities, which are detailed below, the workshop included a series of lectures given by faculty and TAs, to help the participants’ understanding of the topic of AMR, research ethics, and scientific English communication. Herein is a description of the course activities as carried out in 2024.

##### Day 1: Sample Preparation

On Day 1, the students prepared the collected samples (three swabs per group). The swab samples were spread onto agar plates and incubated overnight at 35 °C. The agar plates used were Sheep Blood agar (SBA: non-selective medium that supports the growth of a broad range of bacteria), Mannitol Salt agar (MSA: selective medium for salt-tolerant bacteria such as *Staphylococci*), MSA with 4 µg/mL cefoxitin (MSA/CFX4), DHL agar (non-selective medium), DHL agar with 4 µg/mL cefpodoxime (DHL/CPDX4), and Muller-Hinton agar with 4% NaCl and 6 µg/mL oxacillin (MH + NaCl/MPI4). Students were required to learn the characteristics of each medium. In the afternoon, a Gram staining practice session was conducted using *S. aureus* and *E. coli*.

##### Day 2: Detection of Antibiotic Resistance Genes

Day 2 began with the students observing the bacterial colonies on their agar plates and recording their results, carefully noting the size, morphology, and color of the colonies, texture changes in the agar medium, and the difference in colony numbers among the plates. Following this, PCR was performed to detect antibiotic resistance genes using isolated colonies. This involved detecting the staphylococcal *mecA* gene, responsible for methicillin resistance, from the MSA/CFX4 plate, and Enterobacteriaceae AmpC β-lactamase genes from the DHL/CPDX4 plate. The PCR products of the *mecA* gene and AmpC β-lactamase genes (length variable among species) were submitted to agarose gel electrophoresis. For further experiments, the same colonies tested by PCR were replicated onto MSA/CFX4 and SBA or DHL/CPDX4 and SBA.

##### Day 3: Species Identification and Antibiotic Susceptibility Test

Day 3 was the most demanding in terms of laboratory work. Students performed Gram staining with the bacteria that were tested by PCR using the replicated colonies on SBA plates. They observed differences in cell morphology and were required to recognize the importance of Gram staining in species identification, which is recommended for the proper prescription of antibiotics. Analytical Profile Index (API) kits (bioMérieux, Marcy-l’Étoile, France) are used to identify the bacteria at species level. Although these kits are no longer used in clinical settings in Japan, they remain reliable and convenient research tools that are feasible within a limited timeframe and do not require a series of conventional biochemical tests demanding specialized training or expensive facilities. The API Staph was used to identify *Staphylococcus* species, and the API 20E to identify Enterobacteriaceae. Next, students performed an antibiotic susceptibility test using the Disk Diffusion method according to the Clinical and Laboratory Standards Institute (CLSI) standard procedures [24]. Confirmatory tests for extended-spectrum beta-lactamase (ESBL) production were conducted using the combination disk method, employing cefotaxime and ceftazidime-clavulanate disks. A zone diameter increase of ≥5 mm indicated ESBL production. Detection of AmpC β-lactamases was also performed using the Cefoxitin-Cloxacillin disk diffusion test, where an inhibition zone difference of ≥4 mm indicated AmpC production. Additionally, Minimum Inhibitory Concentration (MIC) was measured using commercially available ‘Dry plates’ for *Staphylococci* and ESBL-producing bacteria. Students prepared an inoculum, added it to MH broth, and inoculated the wells of the Dry plate. They then incubated the plate to observe bacterial growth and determine the MIC values. Later that day, students experienced conventional biochemical tests, such as the catalase test, coagulase test, and cytochrome oxidase test. They also worked with Triple Sugar Iron (TSI) agar to explore the elegant principles for detecting bacterial characteristics.

##### Day 4: Results, Analysis, and Presentation

On Day 4, the final day of the course, the students checked and interpreted the API results using the designated bioMérieux API website (https://apiweb.biomerieux.com/; accessed on 5 September 2025). They also summarized the results of the series of tests that they had performed. Based on the results of the disk diffusion and MIC test, each bacterium was categorized as R (resistance), I (intermediate), or S (susceptible) according to the CLSI criteria. If resistant colonies appeared within the inhibitory zone, this suggested contamination or potential induced resistance, warranting further testing. The MIC results were then analyzed, with MIC defined as the lowest antibiotic concentration that inhibits visible bacterial growth. These results were compared to establish breakpoints and determine susceptibility, i.e., if the MIC was below the breakpoint, the bacteria were susceptible, while an MIC above the breakpoint indicated resistance. The final analysis involved interpreting results from the TSI test, determining whether bacteria could utilize glucose anaerobically, grow under anaerobic conditions, utilize lactose or sucrose, produce gas, or generate hydrogen sulfide. Using the data collected, students worked to identify the bacterial species as accurately as possible, following bacterial identification flowcharts. Finally, the students participated in group discussions and prepared presentations to share summaries of their results. All participants took part in the final presentation and discussion session.

### 2.2. Workshop Evaluation Methodology

Since its inception in 2017, feedback on the workshop has been gathered in the form of short reports written by participants from the University of Tsukuba. These reports, which were written in either Japanese or English, have been used to evaluate the course and to consider ways to improve it. To reflect on the educational merits of Infection Diagnosis Workshop from a student’s perspective for the current study, the reports were collated, read, and analyzed. Qualitative textual analysis was used to identify recurring themes and key insights, both explicit and latent, related to students’ experiences [25], with the aim of providing a comprehensive understanding of their perspectives on the workshop. Thematic analysis was employed to systematically code and categorize the content of the reports, allowing for the identification of patterns across the dataset [26,27,28]. The reports were initially read to familiarize the researcher with the content, then coded to identify the key learning outcomes, and these codes were grouped into broader themes. From 2025 onward, question items were added to the report requirements that were designed to assess changes in students’ understanding of global challenges, multicultural awareness, communication capabilities, and preparedness for future work in international contexts in line with the university’s Social Impact Project goals (see Appendix A). The students gave informed consent for their reports to be utilized for research and publication purposes, and some have been published prior in their original language in the university’s online journal (https://www.md.tsukuba.ac.jp/med-sciences/tjms/; accessed on 27 September 2025). This study was conducted in accordance with the Declaration of Helsinki and was granted an exemption from ethics approval by the Ethics Committee of the Institute of Medicine, University of Tsukuba.

## 3. Results

### 3.1. Results of Thematic Analysis

From the thematic analysis of the students’ reports, six major codes were identified: (1) Awareness and Knowledge; (2) Technical Skills; (3) Cultural Understanding; (4) Cross-Cultural Collaboration; (5) English Language; and (6) Communication Skills. These codes were grouped into three themes: “Knowledge, Awareness, and Technical Skills”, “Cultural Understanding and Cross-Cultural Collaboration”, and “English Language and Communication Skills.” The results of the thematic analysis are shown in Table 1, which includes an explanation of each of the six codes and an example sentence from the students’ essays.

The identified codes and themes provide a comprehensive overview of the majority of sentiments and ideas expressed in their essays. Subsequently, a more in-depth discussion of each theme follows.

#### 3.1.1. Knowledge, Awareness, and Technical Skills

According to the students’ feedback, the Infection Diagnosis Workshop enhanced their knowledge concerning AMR and infectious disease diagnosis. Students indicated that they gained a comprehensive understanding of AMR and its critical importance in global healthcare. Through hands-on experiments and group discussions, they explored the prevalence of AMR, mechanisms of resistance, contributing factors, and broader public health implications. The workshop provided direct experience in diagnostic laboratory techniques, such as pathogen identification, PCR, and antimicrobial susceptibility testing. These new skills expanded the students’ technical toolkit, reinforced their theoretical knowledge, and built confidence in their lab capabilities, preparing them for advanced work in clinical and research settings. The practical experience and understanding of diagnostic workflows were particularly relevant for careers in healthcare, microbiology, or infectious disease research.

Students also practiced analyzing and interpreting their experimental results, honing their critical thinking skills. Group discussions about their findings helped them learn to troubleshoot collaboratively, key skills for any scientific or healthcare role. The hands-on experience allowed students to apply theoretical knowledge to real-world challenges, motivating them to address pressing global health issues. Furthermore, collecting and analyzing samples from the environment and identifying various pathogenic bacteria in those samples gave the students a stark lesson in the consequences of improper antibiotic use and an appreciation for the complexities of antimicrobial stewardship. Engaging in laboratory work and participating in discussions strengthened the students’ problem-solving and analytical skills, enhancing their diagnostic decision-making abilities. This experience cultivated a global perspective on infectious diseases, helping students recognize AMR as a pressing worldwide issue and motivating them to address global health challenges.

#### 3.1.2. Cultural Understanding and Cross-Cultural Collaboration

The Infection Diagnosis Workshop provided a valuable opportunity for students to deepen their cultural understanding, develop their international posture, and cross-cultural collaboration skills. Students interacted closely with peers from various countries, enriching their academic and personal perspectives by understanding different viewpoints on medical science, healthcare practices, ethical considerations, and problem-solving approaches. This overseas experience, working in groups alongside Vietnamese participants to carry out research, highlighted differences in cultural norms and communication styles, helping students learn to navigate social interactions with respect and adaptability. In some years, when our hosting institution has been affiliated with a hospital, the program has included site visits to Vietnamese hospitals. While not a fundamental component of the workshop, these visits have provided students with direct exposure to healthcare practices in Vietnam and shown them the impact of cultural and systemic differences on public health. This awareness has helped students recognize how local practices can influence healthcare outcomes, adding a valuable global health perspective especially for an issue such as AMR. Many students also formed lasting friendships with their international peers, deepening their sense of global citizenship and encouraging them to continue engaging with diverse cultures and academic practices. Moreover, collaborating with peers in a supportive environment reinforced their belief in their abilities, including their ability to contribute within a team. Reflecting on their progress in the reports, the students attest to tangible improvements in their confidence, skills, and cultural adaptability, giving them a sense of accomplishment in their personal and professional growth.

#### 3.1.3. Language and Communication Skills

The students’ reports indicated that the workshop boosted their confidence in communicating in English, especially within an academic and technical context. Initial anxiety around speaking in English, participating in discussions, or adapting to a new environment often led to moments of feeling overwhelmed. However, these struggles eventually transformed into determination. Many noted a sense of accomplishment from participating in group discussions, successfully sharing their ideas, and being understood. Engaging in discussions about complex topics allowed them to practice specialized vocabulary, while repeated exposure to technical English improved their fluency and confidence in discussing scientific subjects. Likewise, preparing and delivering group presentations in English further developed students’ confidence in public speaking. Overcoming an initial anxiety, many reported feeling more comfortable presenting their research to an international audience and a sense of achievement in doing so. Positive experiences in communicating in English motivated them to continue improving their language skills, which will benefit them in future academic, professional, and multicultural interactions.

## 4. Discussion

### 4.1. Educational Reflections

The findings from the thematic analysis of the student essays demonstrate that the Infection Diagnosis Workshop has led to perceived gains in their knowledge, technical skills, cultural understanding, and communication abilities. These outcomes align with the goals of similar education programs that aim to integrate practical, cross-cultural, and communicative experiences into health sciences education. Notably, the hands-on learning aspect of the workshop provided students with an opportunity to deepen their knowledge of AMR and infectious disease diagnostics, much like other experiential learning models, including “One Health” approaches [29,30,31,32]. For example, Fuhrmeister and colleagues discuss a classroom-based initiative called the Prevalence of Antibiotic Resistance in the Environment (PARE) [32]. The PARE program involves students worldwide in systematic environmental surveillance of antibiotic resistance, aiming to pinpoint areas with high prevalence [32]. Such hands-on programs emphasize the value of engaging students in real-world scenarios to solidify their theoretical understanding and build confidence in technical skills. In the Infection Diagnosis Workshop, our students’ exposure to diagnostic laboratory techniques mirrors the experiential learning seen in other medical education initiatives that prioritize the link between theoretical knowledge and practical skill development [12,13,33,34]. The ability to apply these skills in a clinical setting in the future further encourages students in their path towards careers in healthcare and research.

One of the key successes of the workshop has been its inclusive design, bringing together a diverse group of participants from across multiple disciplines within the university. By intentionally inviting students from various fields such as medicine, environmental science, physics, and computer science, the program created a unique environment for interdisciplinary exchange and reflects the holistic, One Health nature of AMR, a global challenge that spans human, animal, and environmental systems. For students from non-biomedical disciplines, such as physics and computer science, participation in laboratory-based AMR training provides direct exposure to the biological, clinical, and public health dimensions of antimicrobial resistance. While the workshop does not explicitly train students in applying their discipline-specific tools during the course, it deepens their understanding of the complexity and scale of AMR as a multidimensional problem. This experiential learning can encourage students to reflect on how knowledge and methodologies from their own fields, such as quantitative modeling, data analysis, or AI approaches, might be applied in future research or professional contexts and explore how their expertise could contribute to AMR-related challenges beyond the immediate scope of the course. In addition, students gained insight into the roles and responsibilities of specialists within a multidisciplinary team, helping them to better understand how their own expertise can contribute to collective problem-solving as a foundation for future research innovation and the development of novel, interdisciplinary solutions to complex global problems like AMR. This experience may also have motivated participants to deepen their knowledge in their respective fields, with a renewed sense of purpose and professional identity. The following student reflection captures how this approach deepened their understanding of complex global health challenges and highlighted the value of collaborative, cross-sector solutions:

One of the things I strongly felt through this workshop was the importance of interdisciplinary collaboration in solving problems. Over the course of the approximately eight-day training, we explored ways to address the issue of antimicrobial-resistant bacteria. When we hear the term “antimicrobial resistance”, it is often perceived as a problem limited to the medical field. However, solving this issue requires connections across various fields such as agriculture, livestock and aquaculture, environmental science, international development, education, and international cooperation. Moreover, this kind of cross-sector collaboration is not only necessary for tackling AMR, but also for addressing many other challenges that we will face in the future. I came to realize that it is important not to remain confined to one’s own field, but to actively engage with other disciplines when tackling complex issues.

This excerpt highlights a key outcome of the course: the realization that complex global health issues like AMR require an integrated, cross-sectoral approach. The students’ reflection aligns closely with the One Health concept, which, as discussed in the Introduction Section, recognizes the interconnectedness of human, animal, and environmental health. By emphasizing the need for collaboration across fields such as medicine, agriculture, environmental science, education, and international cooperation, the students demonstrate a shift toward systems thinking that is essential for addressing global health threats. This insight not only reflects the academic value of the course but also its potential to create meaningful social impact by equipping future professionals with the mindset and tools needed to tackle real-world problems collaboratively and effectively.

An illustrative example of how the workshop encouraged multidisciplinary perspectives can be found in the reflections of a student with an architectural background. Through participation in the hands-on AMR experiments and discussions, the student began to conceptualize antimicrobial resistance beyond a purely biomedical framework, linking it to the built environment and everyday living spaces. Drawing on her prior learning that “the origins of urban planning lie in hygiene,” the student connected architectural design with public health and infectious disease control, particularly in the context of antimicrobial resistance. Exposure to AMR challenges in Southeast Asia further reinforced this perspective, prompting the student to question how “our immediate living environments are involved in this problem.” Notably, the student contrasted disciplinary approaches, observing that “in agricultural studies, we learn to screen for unknown useful microorganisms that match specific objectives, whereas pathogenic microorganism testing involves identifying known pathogen candidates using well-established methods,” a realization that “broadened my horizons to include the medical field.” This example demonstrates how shared experimental experiences encouraged students to reinterpret AMR through their own disciplinary lenses, supporting the development of interdisciplinary awareness and reflective problem framing.

The workshop’s impact on students’ cultural understanding and cross-cultural collaboration is also consistent with similar outcomes from other international education programs [12,13,14,35,36]. For instance, studies of international medical electives have found that working alongside peers from different cultural backgrounds helps students develop an appreciation for diversity and adaptability [36,37,38]. In this workshop, students collaborated with Vietnamese peers, gaining insight into different healthcare practices and the impact of cultural and systemic factors on public health. A systematic review by Lu et al. (2018) revealed that overseas experiences such as these are critical for nurturing a global health perspective and preparing future healthcare professionals with the cultural competencies to work effectively in culturally diverse environments [38]. The value of cross-cultural collaboration was further evident in the formation of lasting relationships and the enhancement of students’ sense of global citizenship, as seen in similar intercultural exchange programs [12,13,14,38,39].

Additionally, the workshop improved students’ perceived English language proficiency and communication skills, particularly in technical and academic contexts. The initial anxiety about using English transformed into motivation as students grew more confident in group discussions and presentations. These results are consistent with previous findings among Japanese healthcare students that international immersion programs enhance language proficiency and boost confidence in non-native speakers [12,13,14,40,41]. The practice of delivering group presentations and using specialized vocabulary not only improved students’ fluency but also prepared them for future multicultural academic and professional settings. Some studies have shown that for Japanese individuals, the challenges of communicating in a second language in an academic or medical context can lead to gains in both language skills and confidence [12,13,14,41,42,43]. On the other hand, the language barrier has the potential to be very stressful in some high-pressure contexts, as noted by Heist et al. (2020) in their study of Japanese international medical graduates who underwent clinical training in the United States [42]. In contrast, the Infection Diagnosis Workshop, held in Vietnam, provides a learning environment where English is used as a lingua franca between non-native speakers [43], creating a more balanced and collaborative approach to language use compared to the potentially one-sided pressure of learning in an English-speaking country.

A limitation of this study is that the analysis relies solely on qualitative reflection reports, which provide rich insight into participant perspectives but do not objectively quantify competency gains. To address this, it would be helpful to complement these reflections with standardized assessments of knowledge and practical skills while maintaining the program’s foundational training focus.

### 4.2. Organizational Reflections

To date, the Infection Diagnosis Workshop has been held eight times, sometimes biannually, with a three-year hiatus for the pandemic. It has been attended by 65 students from the University of Tsukuba and over 100 Vietnamese students. One key aspect of the continued success of the workshop is our partnering with Vietnamese alumni of our university—researchers and academics now working in Vietnam—who provide teaching, on-location support, access to facilities, and the promotion of the workshop among Vietnamese students and colleagues. The University of Tsukuba established its office in Ho Chi Minh City in 2009. Since then, hundreds of Vietnamese students have graduated from our graduate programs. Many of these students, who have returned to Vietnam to take positions in universities or research institutes, have continued their collaboration with the principal investigators of the laboratory they graduated from. As stated earlier, running a course involving experiments with live pathogenic bacteria requires access to laboratory facilities with sufficient equipment and safety level. Having alumni in faculty positions within universities or research institutes can help open doors to such facilities. Maintaining a strong alumni network and keeping relationships with our former students is a key aspect in creating effective and sustainable partnerships and programs such as this workshop.

Alongside the educational benefits for the participating students (discussed above), a secondary objective of the course is to generate interest in our graduate programs among the participating Vietnamese students. By offering an engaging and hands-on workshop experience, we aim to provide these students with a glimpse into the research and learning opportunities available at the University of Tsukuba. This allows us to highlight the unique features of studying in Japan and the advantages of our graduate programs, such as interdisciplinary research opportunities, state-of-the-art facilities, and a strong emphasis on global collaboration. This approach enables participants to not only develop their academic and professional skills but also form a tangible connection to our institution, generating interest in pursuing further education with us. Over the years, our inbound and outbound exchange programs have consistently proven to be an effective strategy for identifying and recruiting promising candidates for our graduate programs [12,13]. These exchanges build lasting relationships with students, alumni, and partner institutions, creating a connection to motivated, well-qualified individuals who are already somewhat familiar with the academic culture and standards of Tsukuba and enhancing the diversity of our graduate student body. This mutually beneficial arrangement ensures that we attract talented individuals while contributing to the professional growth and development of students from partner countries, and, in the case of this workshop, we can raise awareness of the pressing issue of AMR.

### 4.3. Future Directions

To improve the Infection Diagnosis Workshop, two innovations have been made for 2025. Firstly, with the workshop’s support from the university’s Social Impact Project, we have been able to award the participants digital credentials, through the Japan Virtual Campus (JV Campus) (https://www.jv-campus.org/en/; accessed on 20 December 2025) Open Badge System. These digital badges securely record and authenticate students’ skills, achievements, and competencies, functioning as part of an electronic portfolio. They may be used as supplementary credentials on resumes or even professional networking platforms such as LinkedIn. Within our university, the credentials can also support selection processes for scholarships and grant opportunities [44]. To earn the badge, students must complete certain reflective tasks that focus them on global and multicultural issues as well as their own learning and growth (see Appendix A). It is hoped that the award of these digital credentials will increase the participants’ motivation while encouraging them to consider ways in which they can use their skills and education to positively impact society as internationally minded scholars.

Secondly, given the diverse academic backgrounds of the participants, some have expressed a lack of foundational knowledge in the field. To address this, the 2025 workshop introduced expanded pre-workshop assignments aimed at improving student readiness to engage with the topic of AMR. Third- and fourth-year students are assigned to small groups and tasked with preparing a short presentation (approximately 10 min) on an AMR-related topic of their choice, which they must explore though independent research. Students are instructed to verify the accuracy of sources, include references, and present their ideas in a way that would be accessible to junior peers and participants from other disciplines. For students who struggle to select an original topic, a curated list of recent AMR-related articles is provided as an alternative. Example topics include the Global Action Plan on Antimicrobial Resistance published by the WHO [45]; the mechanisms underlying the spread of resistance genes among bacteria [46]; the potential link between anticancer therapy and AMR [47]; and emerging research on novel antimicrobial compounds such as those found in oyster hemolymph [48]. The student presentations will be delivered during the workshop in Vietnam. It is hoped that this pre-workshop assignment will not only enhance student preparedness but also encourage them to take on leadership roles within their groups.

## 5. Conclusions

Overall, the Infection Diagnosis Workshop achieved multiple educational goals that are critical for developing future healthcare professionals. The hands-on experience and exposure to diagnostic laboratory techniques enhanced students’ technical knowledge and confidence, preparing them for roles in healthcare and research. Moreover, the cross-cultural collaboration and English communication practice provided a broader, global perspective and crucial soft skills needed in an increasingly interconnected world. These findings reinforce the value of integrating experiential, cross-cultural, and communicative components into medical and scientific education programs to comprehensively prepare students for the complexities of global healthcare. Furthermore, by bringing together a diverse range of participants from various academic and cultural backgrounds, the workshop encouraged inclusive dialog, expanded the pool of ideas and perspectives, and demonstrated the importance of stakeholder diversity in addressing global health challenges. At the same time, this workshop has helped to forge lasting connections with students, alumni, and partner institutions, attract talented and well-qualified individuals to our graduate programs, support the participating students’ professional growth, and have a direct social impact by raising awareness of the critical issue of AMR.

## Figures and Tables

**Table 1:** Overarching themes and codes identified from student reports.

Theme	Codes and Explanations	Example Statements
Knowledge, Awareness, and Technical Skills	Awareness and Knowledge: This label was applied when students articulated shifts in their understanding, knowledge acquisition, or an expanded perspective on topics that were discussed during the workshop, particularly related to infectious diseases, public health practices, and cultural differences in healthcare. This label is also used for expressions of newfound awareness about the global impact of these issues, highlighting the students’ evolving insight into health as a field influenced by both local and global factors.	“This workshop raised my awareness of antibiotic resistance and the dangers of improper usage. Hearing local researchers’ insights on Vietnam’s situation broadened my perspective on public health practices.”
	Technical Skills: This label captured students’ reflections on acquiring concrete, hands-on abilities that contribute to their proficiency in laboratory environments. It is applied when students explicitly describe learning or performing new laboratory procedures, discuss their feelings of achievement, or mention the impact of these skills on their future career aspirations, especially in healthcare or research settings.	“For the first time, I conducted procedures like Gram staining and PCR. This exposure to diagnostic techniques was thrilling and reinforced my interest in human biology, inspiring me to pursue future research.”
Cultural Understanding and Cross-Cultural Collaboration	Cultural Understanding: This label was used when students reflect on how their exposure to different cultural settings, norms, and interpersonal interactions enriched their educational experience. It encompasses expressions of increased appreciation for cultural diversity, adaptability in a new cultural environment, and the perceived benefits of forming connections with people from different backgrounds.	“Experiencing the local atmosphere and culture and engaging with Vietnamese students enriched my understanding and made the workshop more meaningful beyond academics.”
	Cross-Cultural Collaboration: This label was applied when students describe how engaging with peers from different cultural contexts enhanced their ability to work effectively in a team, improved their problem-solving capabilities, and cultivated a greater appreciation for the diversity of ideas. It emphasizes the value of multicultural teamwork and the growth students experienced as a result of collaborating across cultural boundaries.	“This cross-cultural exchange broadened my global perspective, increased my respect for diversity, and enhanced my ability to collaborate on problem-solving.”
English Language and Communication Skills	English Language: This label is used when students reflect on their use of English, the obstacles they faced, and their motivation to improve their skills to succeed in international academic or professional environments. It encompasses descriptions of using English for presentations, discussions, or scientific contexts, and the resulting awareness of the need to develop language skills further.	“The entire experiment was explained in English, and all communication within the group was also in English, which made me acutely aware of the challenge of using specialized scientific language. Since I want to pursue research in the future, I realized the need to study scientific and medical English more intensively.”
	Communication Skills: This label captured students’ reflections on their efforts to express themselves effectively, the barriers they faced, and the lessons they learned about the importance of communication—both verbal and non-verbal—especially in a collaborative or challenging environment. It also includes instances where students acknowledge growth in their ability to communicate complex ideas or adapt their communication style to suit different audiences.	“Of course, communication wasn’t always easy. I sometimes struggled to express my views and knowledge in a way that was clear and respectful, both in terms of language skills and depth of understanding. However, once my thoughts were understood, they were readily accepted, which reinforced the importance of trying to communicate, even when it’s challenging.”

## Data Availability

The qualitative data generated and analyzed in this study consist of reflective essays written by students who participated in the Infection Diagnosis Workshop between 2017 and 2024. Due to the nature of these data, which contain personally reflective content, full transcripts cannot be made publicly available to protect participant privacy. A selection of essays has been previously published in the University of Tsukuba’s online journal *Tsukuba Journal of Medical Science* (https://www.md.tsukuba.ac.jp/med-sciences/tjms/ accessed on 5 September 2025) and can be accessed there. Further excerpts or aggregated data may be made available from the corresponding author upon reasonable request and with appropriate ethical considerations.

## Abbreviations

The following abbreviations are used in this manuscript:

AI	Artificial Intelligence
AMR	Antimicrobial Resistance
API	Analytical Profile Index
CLSI	Clinical and Laboratory Standards Institute
DHL	Deoxycholate Hydrogen Sulfide Lactose agar
ESBL	Extended-Spectrum Beta-Lactamase
IQR	Interquartile Range
MIC	Minimum Inhibitory Concentration
MSA	Mannitol Salt Agar
MSA/CFX4	Mannitol Salt Agar with 4 µg/mL Cefoxitin
MH + NaCl/MPI4	Muller Hinton agar with 4% NaCl and 6 µg/mL Oxacillin
PCR	Polymerase Chain Reaction
SBA	Sheep Blood Agar
TSI	Triple Sugar Iron

## Appendix A

Items used to guide student report writing in accordance with the goals of the University of Tsukuba’s Social Impact Project.

Understanding Global Issues

1. (Before the course) What motivated you to participate in this activity, and were there any specific global issues or topics you hoped to learn about?
2. (After the course) What topics were discussed during the activity, and how did they relate to global issues, if at all?

Multicultural Awareness

3. (Before the course) Why are you interested in learning about other cultures? Please describe a specific experience you have had in a multicultural environment and explain how it influenced your interest.
4. (After the course) What did you learn or become aware of regarding other cultures through this activity? Were any specific topics related to cultural differences discussed?

Communication Capabilities

5. (Before the course) What specific activities are you currently engaged in to become more comfortable and confident when communicating with people from different cultural or social backgrounds?

(If you have not yet had such experiences, please describe your goals for communication in a multicultural environment.)

6. (After the course) After completing the activity, what actions or initiatives do you plan to take to further develop your communication skills for working with people from different backgrounds or cultures?

Career Design

7. (Before the course) What kind of future career excites and motivates you? Does this career involve opportunities to work overseas or collaborate with international partners?
8. (After the course) Through this activity, what have you learned about the preparation needed to work in a global or international environment in the future?

Engagement and Motivation

9. Did the activity capture and sustain your interest?
10. Do you feel that this activity encouraged or motivated you to pursue further learning in multicultural or international contexts?

## References

[1] World Health Organization Antimicrobial Resistance. https://www.who.int/news-room/fact-sheets/detail/antimicrobial-resistance.

[2] Institute for Health Metrics and Evaluation The Burden of Antimicrobial Resistance (AMR) in Viet Nam. https://www.healthdata.org/sites/default/files/2023-09/Viet_Nam.pdf.

[3] Pessoa Silva C.L. (2020). Global Antimicrobial Resistance and Use Surveillance System (GLASS); Presentation to the Committee on the Long Term Medical and Economic Effects of Antimicrobial Resistance.

[4] Lee A.S., de Lencastre H., Garau J., Kluytmans J., Malhotra-Kumar S., Peschel A., Harbarth S. (2018). Methicillin Resistant *Staphylococcus aureus*. Nat. Rev. Dis. Primers.

[5] Yam E.L.Y., Hsu L.Y., Yap E.P.H., Yeo T.W., Lee V., Schlundt J., Lwin M.O., Limmathurotsakul D., Jit M., Dedon P. (2019). Antimicrobial Resistance in the Asia Pacific Region: A Meeting Report. Antimicrob. Resist. Infect. Control.

[6] Chereau F., Opatowski L., Tourdjman M., Vong S. (2017). Risk Assessment for Antibiotic Resistance in South East Asia. BMJ.

[7] Torumkuney D., Kundu S., Van Vu G., Nguyen H.A., Pham H.V., Kamble P., Lan N.T.H., Keles N. (2022). Country Data on AMR in Vietnam in the Context of Community Acquired Respiratory Tract Infections. J. Antimicrob. Chemother..

[8] Honda H., Goto T., Uehara Y., Takamatsu A. (2023). Promotion of Antimicrobial Stewardship Following Issuance of the Antimicrobial Resistance National Action Plan in Japan. Int. J. Antimicrob. Agents.

[9] Fukuda T., Watanabe H., Ido S., Shiragami M. (2014). Contribution of Antimicrobial Stewardship Programs to Reduction of Antimicrobial Therapy Costs in a Community Hospital. J. Pharm. Policy Pract..

[10] Maeda M., Muraki Y., Kosaka T., Yamada T., Aoki Y., Kaku M., Kawaguchi T., Seki M., Tanabe Y., Fujita N. (2019). The First Nationwide Survey of Antimicrobial Stewardship Programs. J. Infect. Chemother..

[11] Uchida H., Tada T., Tohya M., Sugahara Y., Kato A., Miyairi I., Kirikae T. (2019). Emergence in Japan of a *Klebsiella pneumoniae* Isolate Co-Harbouring blaKPC-2 and rmtB. J. Glob. Antimicrob. Resist..

[12] Ho C.K., Mayers T., Morikawa K., Watanabe Y., Ohniwa R., Saito S., Muratani M., Hasegawa H., Thao D.T.P., Wanandi S.I. (2021). Advanced Topics in Biotechnology and Medicine. J. Med. Engl. Educ..

[13] Mayers T., Purdue B., Ho C.K., Ohneda O. (2015). Teaching Science and Learning English. J. Med. Engl. Educ..

[14] Okamura Y., Ngeabngamsri P., Iwano A., Krikeerati T., Yanagisawa K., Jansirirat T., Ohkoshi M., Shimoda T., Mayers T., Kotruchin P. (2024). A Novel Student-Initiated International Emergency Medicine Competition. Emerg. Care Med..

[15] Marvasi M., Casillas L., Vassallo A., Purchase D. (2021). Educational Activities Supporting the One Health Approach. Antibiotics.

[16] World Health Organization One Health. https://www.who.int/health-topics/one-health#tab=tab_1.

[17] Monath T.P., Kahn L.H., Kaplan B. (2010). One Health Perspective. ILAR J..

[18] American Veterinary Medical Association One Health: A New Professional Imperative. https://www.avma.org/sites/default/files/resources/onehealth_final.pdf.

[19] Nguyen T.T.P., Do T.X., Nguyen H.A., Nguyen C.T.T., Meyer J.C., Godman B., Skosana P., Nguyen B.T. (2022). A National Survey of Dispensing Practice and Customer Knowledge on Antibiotic Use in Vietnam and the Implications. Antibiotics.

[20] Beardsley J., Chambers J.M., Lam T.T., Zawahir S., Le H., Nguyen T.A., Walsh M., Van P.T.T., Van N.T.C., Hoang T.H. (2023). Mapping access to drug outlets in Vietnam: Distribution of drug outlets and the sociodemographic characteristics of the communities they serve. Lancet Reg. Health West. Pac..

[21] Al-Khalaifah H., Rahman M.H., Al-Surrayai T., Al-Dhumair A., Al-Hasan M. (2025). A One-Health Perspective of Antimicrobial Resistance (AMR): Human, Animals and Environmental Health. Life.

[22] University of Tsukuba (2025). Be a Global Start Upper! Social Impact Projects. https://social-impact.projects.tsukuba.ac.jp.

[23] Ministry of Education, Culture, Sports, Science and Technology (MEXT) (2024). Overview of the Social Impact Support Project Through University Internationalization. https://www.mext.go.jp/a_menu/koutou/kaikaku/sekaitenkai/index_00004.htm.

[24] (2018). Clinical and Laboratory Standards Institute (CLSI). Performance Standards for Antimicrobial Disk Susceptibility Tests.

[25] Graneheim U.H., Lundman B. (2004). Qualitative Content Analysis in Nursing Research. Nurse Educ. Today.

[26] Braun V., Clarke V. (2006). Using Thematic Analysis in Psychology. Qual. Res. Psychol..

[27] Squires V., Okoko J.M., Tunison S., Walker K.D. (2023). Thematic Analysis. Varieties of Qualitative Research Methods.

[28] Nowell L.S., Norris J.M., White D.E., Moules N.J. (2017). Thematic Analysis: Trustworthiness Criteria. Int. J. Qual. Methods.

[29] Rabinowitz P.M., Natterson-Horowitz B.J., Kahn L.H., Kock R., Pappaioanou M. (2017). Incorporating One Health into Medical Education. BMC Med. Educ..

[30] Courtenay M., Sweeney J., Zielinska P., Brown Blake S., La Ragione R. (2015). One Health: An Interprofessional Approach. J. Interprof. Care.

[31] Machalaba C., Raufman J., Anyamba A., Berrian A.M., Berthe F.C.J., Gray G.C., Jonas O., Karesh W.B., Larsen M.H., Laxminarayan R. (2021). Applying a One Health Approach. Ann. Glob. Health.

[32] Fuhrmeister E.R., Larson J.R., Kleinschmit A.J., Kirby J.E., Pickering A.J., Bascom Slack C.A. (2021). Combating Antimicrobial Resistance Through Student Research. Front. Microbiol..

[33] Gliddon C.M., Rosengren J.R. (2012). A Laboratory Course for Teaching Research Skills. Biochem. Mol. Biol. Educ..

[34] Orhan T.Y., Sahin N. (2018). Innovative Teaching Approaches in Biotechnology. Educ. Sci..

[35] Morris M., Jones T.D., Rocha M.J.A., Fazan R., Chapleau M.W., Salgado H.C., Johnson A.K., Irigoyen M.C., Michelini L.C., Goldstein D.L. (2006). International Exchange and Medical Curriculum. Adv. Physiol. Educ..

[36] Jacobs F., Stegmann K., Siebeck M. (2014). Promoting Medical Competencies Through Exchange Programs. BMC Med. Educ..

[37] Brouwer E., Driessen E., Mamat N.H., Nadarajah V.D., Somodi K., Frambach J. (2019). International Medical Programmes Design. Med. Teach..

[38] Lu P.M., Park E.E., Rabin T.L., Schwartz J.I., Shearer L.S., Siegler E.L., Peck R.N. (2018). Impact of global health electives on US medical residents: A systematic review. Ann. Glob. Health.

[39] Litzelman D.K., Gardner A., Einterz R.M., Owiti P., Wambui C., Huskins J.C., Schmitt-Wendholt K.M., Stone G.S., Ayuo P.O., Inui T.S. (2017). Transformative Learning Through Global Health. Ann. Glob. Health.

[40] Huffman J., Inoue M., Asahara K., Oguro M., Okubo N., Umeda M., Nagai T., Tashiro J., Nakajima K., Uriuda M. (2020). Identity Development of Japanese Nursing Students. Int. J. Med. Educ..

[41] Chiba Y., Nakayama T. (2016). Cultural Immersion by Japanese Nurses. Jpn. J. Nurs. Sci..

[42] Heist B.S., Torok H.M. (2020). Japanese International Medical Graduates. J. Gen. Fam. Med..

[43] Mathis B.J., Mayers T., Miyamasu F. (2022). English as a Vocational Passport. Educ. Sci..

[44] University of Tsukuba Social Impact Project (2025). Multicultural Co-Learning Meister. https://social-impact.projects.tsukuba.ac.jp/global-start-uppers/en/.

[45] World Health Organization (2015). Global Action Plan on Antimicrobial Resistance.

[46] University of Tsukuba (2022). Mechanism Revealed for Spread of Antibiotic Resistance. Phys.org.

[47] Sharma I., Wilson R.C., Zhu N., Rawson T.M. (2025). Does Anticancer Therapy Directly Contribute to AMR?. Lancet Microbe.

[48] Summer K., Benkendorff K. (2025). Oyster ‘Blood’ and Drug-Resistant Superbugs. InSight+.

